# Effect of HSP25 loss on muscle contractile function and running wheel activity in young and old mice

**DOI:** 10.3389/fphys.2013.00398

**Published:** 2013-12-31

**Authors:** Kimberly A. Huey, Carolyn A. Hilliard, Clayton R. Hunt

**Affiliations:** ^1^Muscle Physiology Lab, Department of Biomedical, Pharmaceutical, and Administrative Sciences, College of Pharmacy and Health Sciences, Drake University, Des MoinesIA, USA; ^2^Department of Radiation Oncology, Washington University School of Medicine, St LouisMO, USA

**Keywords:** heat shock protein 25, skeletal muscle, muscle force, fatigue, running wheel, contractile protein

## Abstract

Aging is associated with an adverse decline in muscle function, often manifesting as decreased strength and increased muscle fatigability that negatively affects the overall health of the elderly. Heat shock proteins (HSPs), a family of stress inducible proteins known to protect cells from damage, are highly induced in muscle cells following exercise, but both basal and inducible levels decline with age. Utilizing young and old mice lacking HSP25 (*H*sp25^−/−^) we tested the hypothesis that HSP25 is required to maintain normal muscle function and that age related decreases in HSP25 directly contribute to declining muscle function. Running wheel distances over 14 days for young *H*sp25^−/−^ mice were significantly lower than for the corresponding *H*sp25^+/+^ genotype (81238 vs. 33956 AUC, respectively). While older groups both ran significantly less than young groups, in aged mice HSP25 loss did not lead to any additional decrease. Significantly lower myofibrillar (contractile) protein levels in young *H*sp25^−/−^ vs. *H*sp25^+/+^ (15.7 ± 0.2 vs. 13.4 ± 0.3 mg/mg muscle) mice suggests HSP25 loss was associated with greater muscle breakdown during voluntary wheel running. *In vivo*, plantarflexor maximal isometric force was significantly decreased in aged vs. young mice, but the loss of HSP25 had no effect on either group. However, plantarflexor fatigability over 10 contractions was significantly higher in young *H*sp25^−/−^ vs. *H*sp25^+/+^ mice (59 ± 3 vs. 49 ± 4% of initial force, respectively) but no similar effect of genotype was detected in the older groups. There was no difference in muscle caspase-3 activity between *H*sp25^−/−^ and *H*sp25^+/+ mice^, whether young or old, but there was a significant genotype independent increase in activity with age. Overall, the results suggest that the absence of HSP25 primarily contributes to muscle fatigue resistance, rather than maximal force production, and that this effect is most evident in young compared to older mice.

## Introduction

Existing evidence suggests that heat shock proteins (HSPs) are involved in normal muscle function and adaptation to various stressors (Welsh and Gaestel, 1998; Nishimura and Sharp, 2005). Within the family of HSPs, the small HSP25 protein encoded by the *Hsp25* gene (HSP27 and *Hsp27* in humans) is abundantly expressed in human (Morton et al., 2009), rat (Golenhofen et al., 2004; Huey et al., 2004, 2010; Larkins et al., 2012), and mouse (Huey et al., 2010) skeletal muscle and responds to changes in muscle loading and activity (i.e., exercise, overload). For example, increases in muscle contractile activity, such as with 2 weeks of voluntary wheel running, significantly increases HSP25 levels in active mouse skeletal muscle (Huey and Meador, 2008). In addition, 1 week of functional muscle overload, an *in vivo* model of muscle hypertrophy, dramatically increases muscle loading and activity and is associated with increased HSP25 protein expression in slow and fast mouse and rat muscles (Huey, 2006; Huey et al., 2007, 2010). Stimulated resistance training for 4.5 weeks in rats also leads to elevated HSP25 expression and in young rats there was a significant correlation between isometric muscle force and HSP25 content (Murlasits et al., 2006).

Various functions for skeletal muscle HSP25 have been suggested including stabilization of muscle structure and repairing damaged muscle proteins (Koh, 2002; Koh and Escobedo, 2004; Paulsen et al., 2007, 2009) as well as reducing apoptosis (Concannon et al., 2003; Beere, 2005). Some protective functions of HSP25 are suggested by its translocation from the soluble (cytosolic) to insoluble (myofibrillar) fraction and binding to cytoskeletal structures such as Z-disk proteins (Koh and Escobedo, 2004; Paulsen et al., 2007, 2009). Interestingly, some muscle damage may be necessary for HSP27 up-regulation as it was reported that HSP72, but not HSP27, was increased in human skeletal muscle following a bout of non-damaging exercise (Morton et al., 2006). In contrast, in a model of muscle damage, 30 min of downhill running was associated with increases in HSP27 in human quadriceps 24 h after exercise (Feasson et al., 2002). The apoptotic functions of HSP25 are suggested by reports that HSP25 has been shown to reduce apoptosis in cultured muscle C2C12 cells (Jiang et al., 2005; Vasconsuelo et al., 2010) and HSP25 has been shown to inhibit both the intrinsic and extrinsic apoptotic cell death pathway at several points including caspase-3 activation (Welsh and Gaestel, 1998), which is strongly implicated in accelerated breakdown of muscle contractile proteins (Du et al., 2004; McClung et al., 2007). *In vivo*, in hypertrophying quail muscle, increases in HSP25 are associated with reductions in apoptosis compared to muscles without increased HSP expression (Siu and Alway, 2005).

Aging is associated with a well-documented reduction in muscle strength and endurance referred to as sarcopenia (Karakelides and Sreekumaran Nair, 2005). Aging is also associated with decreased basal and stress inducible HSP levels, suggesting that inadequate HSP levels may play an important role in age-related declines in muscle function. In adult mice, but not aged mice, HSP25 is significantly increased in muscle after a single bout of isometric exercise (Vasilaki et al., 2006). Similarly, in response to a 4.5 week resistance training program, muscle HSP25 levels were 2-fold greater in young (3 months) as compared to older (30 months) rats (Murlasits et al., 2006). Age-related declines in the up-regulation of another member of the HSP family, HSP70, have been shown following isometric muscle contractions (Vasilaki et al., 2002). Additionally, transgenic over-expression of HSP70 in the mouse extensor digitorum longus muscle was associated with maintenance of specific tension and improved recovery from contractile activity in older mice (McArdle et al., 2004). Thus, since HSPs may help preserve muscle integrity and assist in muscle repair, a reduced basal level or HSP response in aging muscle may be one factor contributing to declining muscle function and the failure of aged muscle to adapt rigorously to increases in muscle activation and loading.

Based upon the purported importance of HSP25 in muscle contractile function and adaptability to an exercise stressor, we utilized *H*sp25^+/+^ and *Hsp*25^−/−^ mice to specifically test if the absence of HSP25 compromises muscle force production and fatigability in young or old mice. Results from the experiments identified a direct relationship between HSP25 and normal muscle endurance, but not maximal force production, in young mice that changes with age.

## Materials and methods

### Animals

All procedures were approved by the Drake University Institutional Animal Care and Use Committee or the Washington University Institutional Animal Care and Use Committee and followed the American Physiological Society Animal Care Guidelines. Deletion of the *Hsp25* gene (*Hspb1*) was done by targeted replacement of a BamH1/Avr2 genomic fragment spanning the methionine initiation site and the first exon of the gene with PGK-neo. Recombinant embryonic stem cells (SCC10), identified by Southern blot analysis, were injected into C57Bl/6 blastocysts and germline transmitted offspring identified by tail DNA analysis. Offspring of heterozygous *H*sp25^+/−^ crosses yielded standard size litters with Mendelian gene segregation. Since no fertility or other obvious health defects were observed, homozygous *H*sp25^−/−^ mice were maintained by in-crossing such that wild-type and *H*sp25^−/−^ mice were of a mixed 129X1/SvJ/C57Bl/6 background. Embryonic fibroblasts were genotyped and analyzed by Western blot analysis to confirm that the *H*sp25^−/−^ genotype correlated with HSP25 protein loss.

Young adult male (3–4 months) or aged (22–24 months) *H*sp25^+/+^ or *H*sp25^−/−^ mice were divided into 4 groups: (1) Young force measurement (*n* = 8), (2) Old force measurement (*n* = 8), (3) Young running wheel activity (*n* = 10), and (4) Old running wheel activity (*n* = 12). The mice in the force measurement groups were used as sedentary controls to quantify the muscular HSP25 protein response to 2 weeks of running wheel activity.

### Running wheel activity

Mice were individually housed in cages equipped with running wheels equipped with magnetic reed switches (Respironics, MiniMitter) which were connected to electronic counters. Twenty-four hour access to the wheels was allowed and wheel revolutions were recorded every 24 h during the light cycle.

### Force measurements

Hind-limb plantarflexor isometric force-production was measured as previously described by our laboratory (Meador and Huey, 2009). Briefly, mice were anesthetized with a ketamine/xylazine (40/3 mg/ml) cocktail which was administered 0.1 ml per 25 g body weight i.p., and the sciatic nerve was exposed through the popliteal fossa in order to electrically stimulate the nerve and elict contraction of the plantarflexors. The hindfoot was placed on a footplate attached to a servomotor able to measure applied torque and control ankle rotation (305C-LR, Aurora Scientific). The sciatic nerve was carefully placed on a stimulating dual hook electrode (Grass Technologies part # F-ES-48) connected to a square wave stimulator (A-M Systems) and stimulated at 200 Hz for 1.5 s with a pulse duration of 0.5 ms to evoke a maximum-force contraction. The nerve was kept moist with mineral oil warmed to ~37°C. The servomotor was set to maintain position, ensuring that the contraction was isometric. With 5-s delays between stimulations, the contraction was repeated 9 additional times to examine muscle fatigability. As a measure of fatigue, the 10th contraction was compared to the maximum contraction and expressed as a percentage of maximal force. For all animals, the maximum contraction was the initial contraction. Force was normalized to body weight to compare among conditions. The choice to normalize to body weight rather than muscle weight was based upon utilization of the *in vivo* footplate system in which it is not possible to accurately determine the exact muscle mass contributing to the final force. However, normalization of force to gastrocnemius/soleus complex mass produced similar final results and thus would not affect data interpretation.

### Tissue collection

Immediately after the force measurements or after 14 days of voluntary running, the mice were euthanized by carbon dioxide inhalation followed by cervical dislocation and hindlimb muscles were quickly isolated and immediately frozen on dry ice and stored at −80°C until analysis. For mice in the force studies, the non-stimulated limb muscle was used for protein analyses to avoid any potential confounding factors resulting from the acute muscle testing.

### Western blotting

Western blots were performed to analyze muscle expression of HSP25. Gastrocnemius muscle samples were mechanically homogenized in a buffer containing 50 mM Tris-HCl (pH 7.8), 2 mM potassium phosphate, 2 mM EDTA, 10% glycerol, 1% Triton X-100, 1 mM dithiothreitol, 3 mM benzamidine, 1 mM sodium orthovanadate, 10 mM leupeptin, 5 mg/ml aprotinin, and 1 mM 4-[(2-aminoethyl) benzenesulfonyl fluoride], and supplemented with protease and phosphatase inhibitors (Sigma). Homogenates were immediately centrifuged at 12,000 g at 4°C for 12 min, after which the supernatant—the detergent soluble fraction—was removed. Protein concentrations were determined by Bradford assay (Pierce) using a bovine serum albumin (BSA) standard curve and immediately stored at −20°C for subsequent use in western blotting. Thirty μ g of total protein was boiled in loading buffer for 5 min at 95°C. Proteins were separated by sodium dodecylsulfate–polyacrylamide gel electrophoresis (SDS–PAGE) (12.5%), transferred to PVDF membranes (Millipore), and equal loading was confirmed by Ponceau-S staining. Only gels with equal loading were used for subsequent antibody staining and analysis. After Ponceau-S staining, membranes were blocked for a minimum of 1 h in Tris-buffered saline (TBS) with 5% non-fat milk. After blocking, the blots were incubated overnight in an Hsp25 polyclonal antibody (Enzo Life Sciences ADI-SPA-801) at 1:5000 or an actin polyclonal antibody (Enzo Life Sciences ADI-CSA-400) at 1:1000 in TBS w/2.5% BSA. After primary antibody incubation, blots were washed three times with TBS/0.15% Tween (TBS-T), incubated in anti-rabbit IgG peroxidase-linked secondary antibody (GE Life Sciences NA934) at 1:5000 for 1 h. After the secondary antibody, the membranes were washed 3 times in TBS-T, developed using enhanced chemiluminescence (GE Healthcare). A Biorad ChemiDoc MP Imaging System and Image Lab Version 4.0 were used for capture, detection, and analysis of images.

### Myofibrillar protein assay

All procedures for the myofibrillar extractions were performed on ice as previously described (Thomason et al., 1987). A 40–60 mg gastrocnemius muscle sample was cut from the frozen muscle and quickly weighed on a balance. The weighed samples were homogenized in 20 volumes of a 250 mM sucrose buffer (250 mM sucrose, 100 mM KCl, 5 mM EDTA, and 10 mM Tris, pH 6.8). Extracts were centrifuged at 3000 g for 5 min, and the supernatant was discarded. The pellet was suspended in 10 volumes of a 0.5% Triton X-100 solution (150 mM KCl, 0.5% Triton X-100, and 10 mM Tris, pH 6.8), which is a modification of the solution described by Solaro et al. (1971). The suspension was homogenized and centrifuged at 3000 g for 10 min. The resulting pellet was rinsed with 10 volumes of wash buffer (150 mM KCl and 20 mM Tris, pH 7.0) and then suspended in 10 volumes of storage buffer. Protein concentration was determined with the Bradford protein assay using BSA for the standard curve.

### Caspase-3 activity

Caspase-3 activity was measured in muscle supernatants with the Caspase-Glo Assay System (Promega) accordingly to the manufacturer's instructions. Briefly, 50 ul of the Caspase Glo buffer was added to 50 ul of muscle tissue supernatant in a 96 well plate and incubated at room temperature for 60 min. Luminescence was measured using a Biotek synergy microplate reader. Background activity levels, based on measurements in homogenization buffer only, were measured and subtracted from values in the muscle tissue supernatants. All values were normalized to total protein content. Standard curves were used to determine the linearity of the responses.

### Statistical analyses

The four groups were compared on the following variables: (1) maximal isometric muscle force, (2) muscle fatigability, (3) myofibrillar protein levels, and (4) running wheel distance (total area under the curve). Each of 2 genotypes (*H*sp25^+/+^ or *H*sp25^−/−^) and 2 age groups (young or old) were statistically analyzed with a 2 × 2 factorial arrangement of treatments in a randomized complete block design. This permitted statistical evaluation of the main effects of genotype (*H*sp25^+/+^ vs. *H*sp25^−/−^) and age (young vs. old) and their interaction. Young and old *H*sp25^+/+^ mice under sedentary conditions or after 2 weeks of RW activity were compared on HSP25 skeletal muscle protein levels. This permitted statistical evaluation of the main effects of activity (sedentary vs. running wheel activity) and age (young vs. old) and their interaction. Two-Way ANOVA were performed to assess main effects and interactions and if significance was found, the Bonferroni *post-hoc* test was used to determine the source of the difference. The decline in force over 10 contractions was analyzed with non-linear regression analysis. All analyses were performed with GraphPad Prism 5.0 (San Diego, CA) with the significance level set at *p* < 0.05.

## Results

### Voluntary running wheel activity

**Figure 1:**
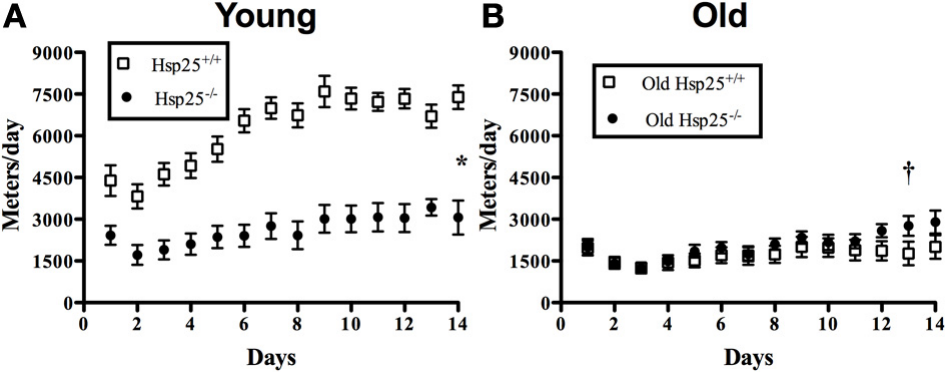
**Average daily running wheel distance in young (A) and old (B) *H*sp25^+/+^ and *H*sp25^−/−^ mice**. Total area under the curve was calculated over 14 days and compared among groups. ^*^Significantly different from young *H*sp25^+/+^ (*P* < 0.0001), ^†^significant main effect of age (*P* < 0.0001). Data are presented as means ± SE, *n* = 10 for young groups and *n* = 12 for old groups.

There was a significant main effect of genotype (*P* < 0.0001) and age (*P* < 0.0001) and a significant interaction of genotype and age (*P* < 0.0001) on running wheel activity measured over 14 days (Figure 1A). In young groups, running wheel activity was significantly lower in *H*sp25^−/−^ as compared to *H*sp25^+/+^ mice. In old groups, there were no significant differences between genotypes (Figure 1B). In *H*sp25^+/+^ mice, aging was associated with significantly lower running wheel activity over 14 days, while the decreased running activity of young *H*sp25^−/−^ mice nearly matched the lower levels observed in aged mice of either genotype.

### Muscle force and fatigability

**Figure 2:**
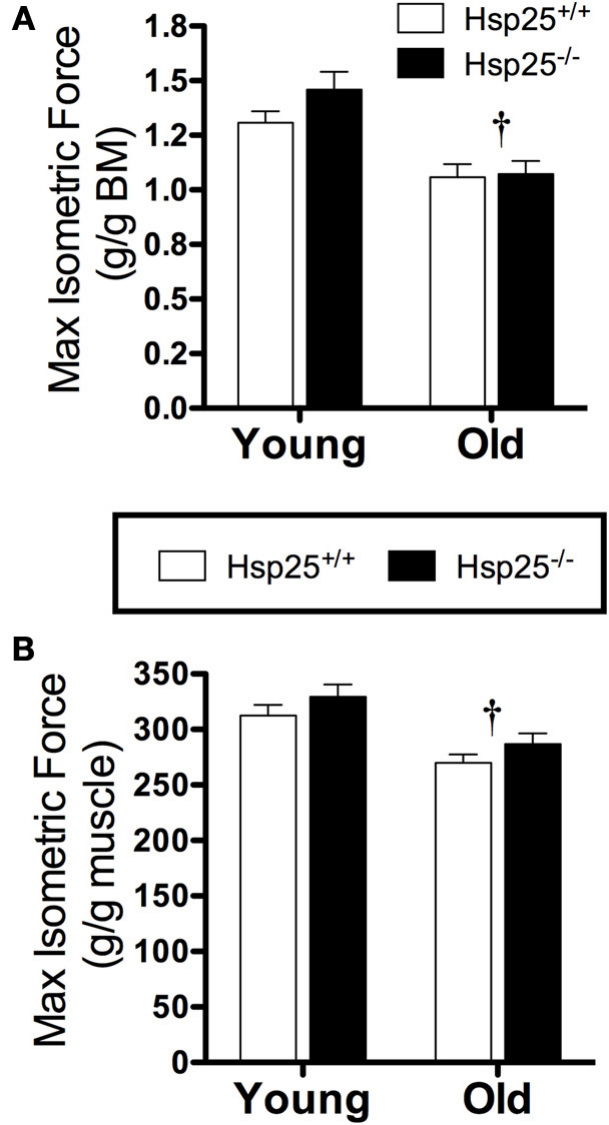
**Average maximal isometric plantarflexor force normalized to body mass (BM) (A) or gastrocnemius muscle mass (B) in young and old *H*sp25^+/+^ and *H*sp25^−/−^ mice**. ^†^Significant main effect of age (*P* < 0.0001 and *P* = 0.0002, respectively). Data are presented as means ± SE, *n* = 8 for all groups.

**Figure 3:**
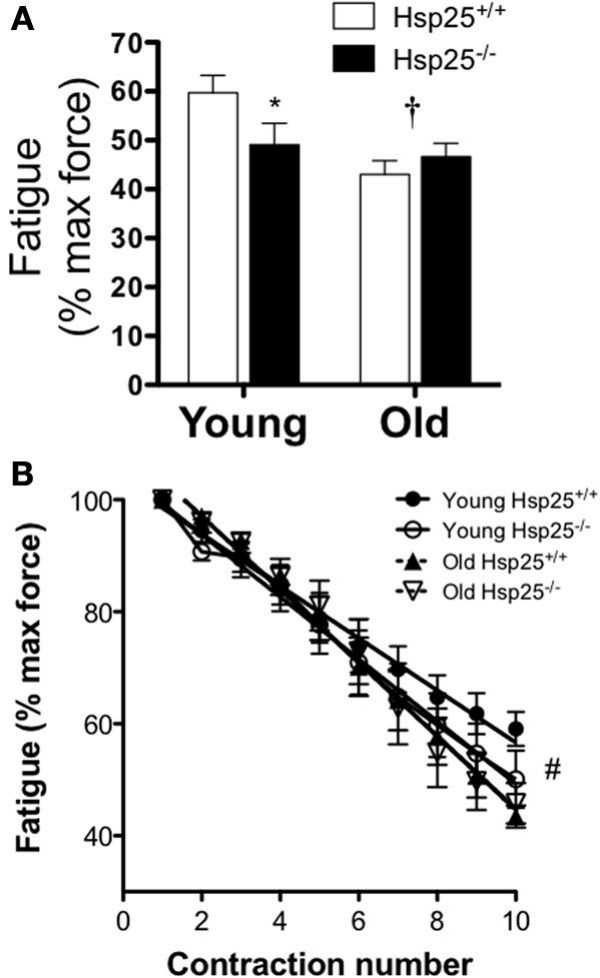
**Average plantarflexor fatigability (A) and rate of force decline (B) in young and old *H*sp25^+/+^ and *H*sp25^−/−^ mice**. Fatigability was calculated as the % of maximal isometric force after 10 contractions with a contraction every 5 s. **(B)** Average rate of force decline shows the % of maximal isometric force for all 10 contractions. ^*^Significantly different from young *H*sp25^+/+^ (*P* = 0.046), ^†^significant main effect of age (*P* = 0.0093), ^#^all groups significantly different from young *H*sp25^+/+^ (*P* < 0.0001). Data are presented as means ± SE, *n* = 8 for all groups.

There was a significant main effect of age on maximal isometric force normalized to body weight (Figure 2A) or muscle mass (Figure 2B). In both genotypes, lower maximal isometric force normalized to body weight (*P* < 0.0001) or muscle mass (*P* = 0.0002) was observed in the older groups compared to the younger groups (*P* < 0.0001) but the absence of HSP25 had no significant effect on either age group. In contrast, there was a significant main effect of age (*P* = 0.0093) and a significant interaction of genotype and age (*P* = 0.0466) on muscle fatigability (Figure 3A). Aging was associated with significantly greater muscle fatigability. The significant interaction shows that genotype has different effects in young vs. old mice. Specifically, in young groups, *H*sp25^−/−^ mice exhibited greater fatigability (Figure 3A) and a greater rate of decline in force over 10 contractions than did *H*sp25^+/+^ mice (*P* < 0.0001) (Figure 3B). Given the short time course of the fatigue measurements (50 s), this outcome likely results from basal level HSP25 loss and not the inability to induce HSP25, since the interval is too short to allow for significant new protein synthesis.

### Body and muscle mass and total myofibrillar proteins

#### Muscle force groups

**Figure 4:**
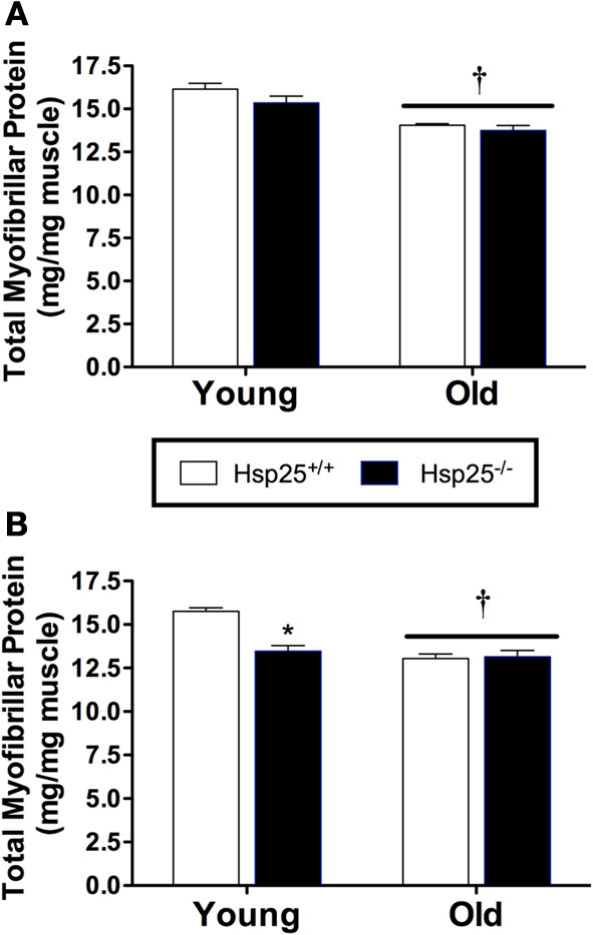
**Average gastrocnemius total myofibrillar protein levels in young and old *H*sp25^+/+^ and *H*sp25^−/−^ mice following muscle contractile measurements (A) or 2 weeks of running wheel activity (B)**. ^*^Significantly different from young *H*sp25^+/+^ (*P* < 0.0001), ^†^significant main effect of age (*P* = 0.0009). Data are presented as means ± SE, *n* = 8 for muscle force groups and *n* = 10 for running wheel groups.

Average body mass was significantly higher in the old compared to young mice independent of genotype (*P* < 0.0001) (Table 1). Gastrocnemius muscle mass and gastrocnemius mass normalized to body mass were significantly lower in the old compared to young mice independent of genotype (*P* < 0.0001) (Table 1). Total myofibrillar protein levels in the gastrocnemius were significantly lower in the old compared to young mice independent of genotype (*P* < 0.0001) (Figure 4A).

**Table 1:** **Final body mass, muscle mass, and muscle mass normalized to body mass for young and old *H*sp25^+/+^ and *H*sp25^−/−^ mice in muscle function and running wheel groups**.

		***Hsp25*^+/+^**	***Hsp25^−/−^***
**BODY MASS (g)**			
Muscle function	Young	23.9 ± 0.4	24.8 ± 0.3
	Old	31.2 ± 0.4^*^	32.2 ± 0.6^*^
Running wheel	Young	24.2 ± 0.2	23.7 ± 0.2
	Old	30.6 ± 0.7^*^	31.8 ± 0.5^*^
**GASTROCNEMIUS MASS (mg)**			
Muscle function	Young	124 ± 3	124 ± 2
	Old	106 ± 2^*^	111 ± 3^*^
Running wheel	Young	119 ± 2	115 ± 2
	Old	102 ± 3^*^	101 ± 3^*^
**GASTROCNEMIUS MASS (mg)/BODY MASS (g)**			
Muscle function	Young	5.0 ± 0.15	4.9 ± 0.18
	Old	3.4 ± 0.09^*^	3.4 ± 0.06^*^
Running wheel	Young	5.2 ± 0.03	5.1 ± 0.07
	Old	3.3 ± 0.05^*^	3.2 ± 0.09^*^

Values are mean ± s.e.m.; n = 8 for muscle function groups and n = 12 for running wheel groups

* significantly different from young group, p < 0.05.

#### Running wheel groups

Average initial body weights for running wheel groups were 24.1 ± 0.3, 30.6 ± 0.6, 23.7 ± 0.4, and 31.7 ± 0.60 g for young and older *H*sp25^+/+^ and *H*sp25^−/−^ mice, respectively, and were significantly higher in the older mice (*P* < 0.0001). Body mass was unchanged with running wheel activity and average final body mass was significantly higher in the old compared to young mice independent of genotype (Table 1). Gastrocnemius muscle mass and gastrocnemius mass normalized to body mass were significantly lower in theold compared to young mice independent of genotype (*P* < 0.0001) (Table 1). For total myofibrillar protein levels in the gastrocnemius there were significant main effects of age (*P* < 0.0001) and genotype (*P* = 0.0009) and a significant interaction of age and genotype (*P* = 0.0004) (Figure 4B). In both genotypes, myofibrillar protein levels were significantly lower in the old compared to young mice. In the young mice, myofibrillar proteins were significantly lower in *H*sp25^−/−^ compared to *H*sp25^+/+^ mice (Figure 4B) and were similar to those in old mice of either genotype.

### HSP25 protein levels

**Figure 5:**
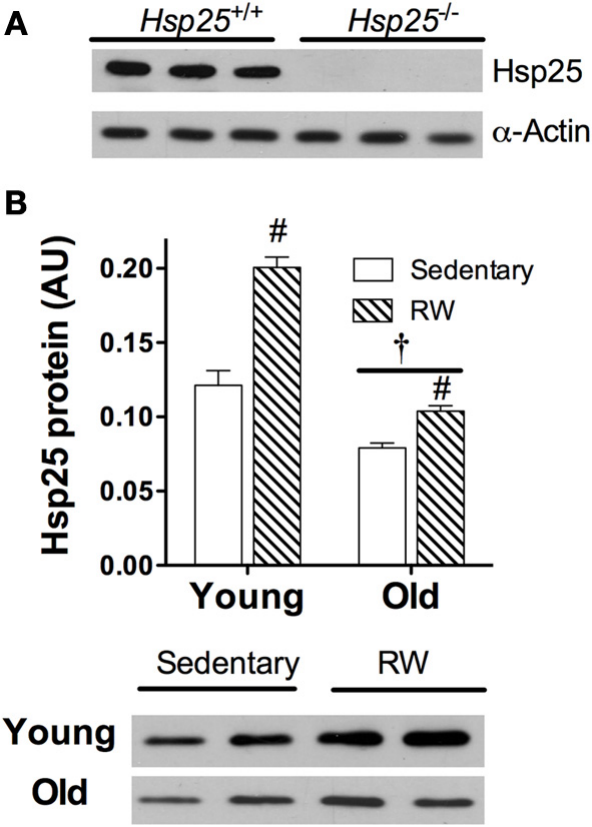
**(A)** Gastrocnemius muscle HSP25 and actin protein levels in *H*sp25^+/+^ and *H*sp25^−/−^ mice verifying the absence of HSP25. **(B)** HSP25 protein levels in young and old *H*sp25^+/+^ mice under sedentary conditions or following 14 days of running wheel activity. ^#^Significantly different from sedentary (*P* < 0.0001), ^†^significant main effect of age (*P* < 0.0001). Representative western blot showing HSP25 in sedentary and running wheel young and old *H*sp25^+/+^ mice. Data are presented as means ± SE, *n* = 8 for all groups.

The absence of HSP25 in the *H*sp25^−/−^ mice was verified by Western blot analysis of gastrocnemius muscle protein (Figure 5A). In sedentary *H*sp25^+/+^ mice, aging was associated with a significant ~35% loss of HSP25 protein in gastrocnemius muscle compared to young mouse muscle (*P* < 0.0001). The levels of HSP25 protein in young *H*sp25^+/+^ gastrocnemius after 2 weeks of running wheel activity was significantly higher (nearly 2-fold) than sedentary mice (*P* < 0.0001) (Figure 5B). In contrast, in aged *H*sp25^+/+^ mice there was only a slight increase in gastrocnemius HSP25 protein levels between running wheel and sedentary mice (Figure 5B).

### Caspase-3 activity

**Figure 6:**
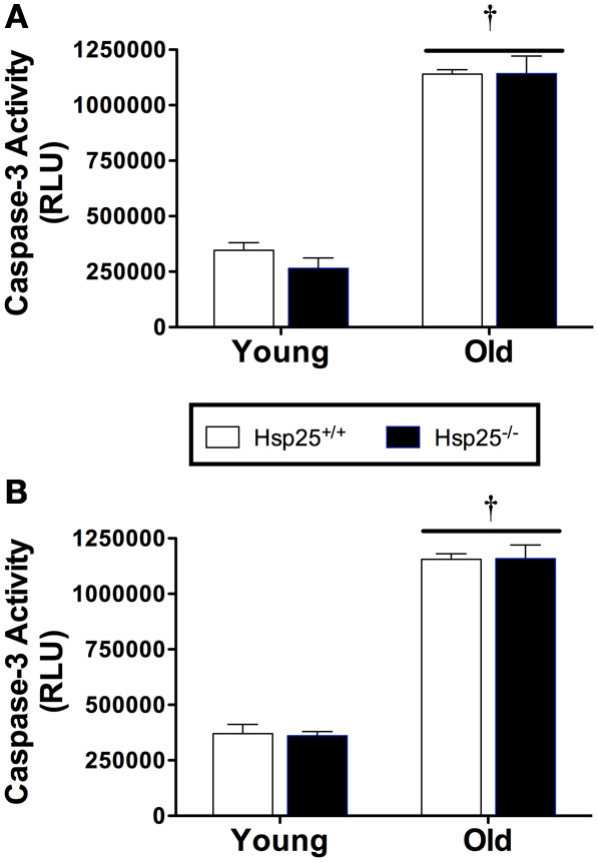
**Average caspase-3 activity (RLU, relative light units) in young and old *H*sp25^+/+^ and *H*sp25^−/−^ mice following muscle contractile measurements (A) or 2 weeks of running wheel activity (B)**. ^†^Significant main effect of age (*P* < 0.0001). Data are presented as means ± SE, *n* = 8 for muscle force groups and *n* = 10 for running wheel groups.

There was a significant main effect of age (*P* < 0.0001) on caspase-3 activity in the gastrocnemius muscle in both the force (Figure 6A) and running wheel groups (Figure 6B). In both genotypes, lower caspase-3 activity was observed in the younger groups compared to the older groups, but the absence of HSP25 had no significant effect on caspase-3 activity in either group.

## Discussion

These novel findings support an important *in vivo* role for HSP25 in skeletal muscle function especially in response to chronic, physiologically relevant increases in muscle activity such as occurs during wheel running. Our lab has previously reported that 2 weeks of voluntary running wheel activity is associated with significant increases in HSP25 in active muscles (Huey and Meador, 2008). The finding that young *H*sp25^−/−^ mice ran significantly less than young *H*sp25^+/+^ mice supports the functional importance of HSP25 during adaptation to chronic increases in muscle activity. Potentially putative functions of HSP25 such as maintaining myofibrillar integrity may be important for muscle structure and function during long periods of increased muscle activity. If muscle damage is a stimulus for HSP25/27 expression (Feasson et al., 2002; Morton et al., 2006) or translocation to cytoskeletal elements (Koh and Escobedo, 2004; Paulsen et al., 2007, 2009), the inability of *H*sp25^−/−^ mice to utilize these responses may contribute to greater muscle damage and consequently reduced ability and/or desire to run. The finding that total contractile (myofibrillar) proteins were significantly lower in *H*sp25^−/−^ muscle compared to *Hsp*25^+/+^ after 14 days of wheel running suggests a reduced ability of *H*sp25^−/−^ muscle to recover from higher levels of activity and likely greater susceptibility to muscle breakdown. While the greater running wheel activity over 2 weeks in the *H*sp25^+/+^ compared to the *H*sp25^−/−^ mice could have stimulated increased muscle protein synthesis, no load running wheel activity is not generally associated with increases in muscle mass or strength (Allen et al., 2001; Meador and Huey, 2011). Furthermore, in the current study, the similar myofibrillar protein levels in *H*sp25^+/+^ mice in the force and running wheel groups shows that running activity did not simulate muscle protein synthesis compared to sedentary conditions.

The finding that aged mice, independent of genotype, ran significantly less than young mice was not unexpected since aging is generally associated with reduced muscle strength and endurance. With aging, the significant loss of contractile protein levels is one likely contributor to this lower running activity. One potential mechanism contributing to the lower levels of contractile proteins in aged mice is the significant increase in caspase-3 activity, since its activation is associated with muscle protein breakdown (Du et al., 2004; McClung et al., 2007). *In vivo*, our lab previously reported that the loss of HSP25 was significantly correlated with increased caspase-3 activity in atrophying rat plantaris (Huey et al., 2008). However, the atrophy model, spinal cord transection, utilized in those experiments clearly differs from age-associated atrophy. The present *in vivo* findings suggest that in normal muscle the loss of HSP25 does not impact caspase-3 activity regardless of age. However, the substantial increase in caspase-3 activity in old compared to young mice, independent of genotype, clearly supports the role of caspase-3 in age-associated muscle atrophy.

Running wheel activity involves numerous physiological systems (e.g., cardiorespiratory) that could be sensitive to the absence of HSP25. We previously reported that 2 weeks of running wheel activity was not associated with increases in HSP25 in cardiac muscle, in contrast to the ~2-fold increases in active skeletal muscle (Huey and Meador, 2008). It is not yet possible to identify all tissue-specific contributions of HSP25 to running activity, but its importance in skeletal muscle during whole body exercise is supported in part by our *in vivo* contractile data. Specifically, in young mice the absence of HSP25 was associated with increased muscle fatigability over 10 isometric contractions. While the type of fatigue experienced during voluntary no load running activity is likely mechanistically different from that of serial high force contractions, both results suggest an overall increase in muscle fatigability in the absence of HSP25. An association between HSP25 and muscular endurance is also indirectly suggested by the higher levels of HSP25 expression in slow, oxidative muscles compared to fast, glycolytic rat muscles (Inaguma et al., 1993; Golenhofen et al., 2004; Huey et al., 2004). Specifically, running wheel activity may rely more on slow, oxidative than fast, glycolytic fibers and HSP25 has been shown to contribute to protection against oxidative stress in cultured C2C12 muscle cells (Escobedo et al., 2004). Further, maximal isometric force production which would rely more on fast, oxidative fibers was not significantly affected by genotype, but was significantly lower in old mice independent of genotype. This significant loss in muscle force production with aging is likely due to part to lower levels of contractile proteins which are the major contributors to force production. Taken together with the running data, HSP25 appears to have a greater role in muscular endurance (repeated contractions of lower intensity) compared to absolute muscle strength (force production in a single maximal contraction).

Some evidence suggests that reductions in basal HSP25 levels in skeletal muscle as well as reduced up-regulation of HSP25 in response to a physical stressor plays a role in age-related declines in muscle function. While resistance training increased HSP25 in the muscles of both young (3 months) and older (30 months) rats, there was a 2-fold greater response in young mice (Murlasits et al., 2006). The HSP25 response in aged muscle is also attenuated following only a single bout of isometric exercise (Vasilaki et al., 2006). The present study is first to report of the effects of age on the skeletal muscle HSP25 response to aerobic exercise (voluntary wheel running). In *H*sp25^+/+^ mice, basal levels of HSP25 were ~35% lower in old compared to young mice and in old *H*sp25^+/+^ mice HSP25 induction by running wheel activity was significantly blunted. This is in contrast to young muscle in which previous findings (Huey and Meador, 2008), and the current experiments demonstrate that running wheel activity is associated with much higher levels of HSP25 in active muscles. This novel running wheel data support in part that age-related reductions in the ability to up-regulate HSP25 in response to a stressor may negatively impact muscle function with aging. Specifically, while the differences in running activity between young *H*sp25^+/+^ and *H*sp25^−/−^ mice were dramatic, there were no differences between genotypes in aged groups. Hypothetically, a reduced ability of old *H*sp25^+/+^ mice to up-regulate HSP25 in response to the running stressor makes them more similar to *H*sp25^−/−^ mice while the differences between HSP25 levels in young groups would be expected to be greater.

Apoptosis is critical in maintaining tissue integrity, especially in proliferative tissues, and up-regulation of apoptotic pathways in skeletal muscle constitutes a major mechanism by which aged muscle mass declines (Marzetti and Leeuwenburgh, 2006). HSP25 has been shown to reduce apoptosis in cultured muscle C2C12 cells by associating with caspase-3 (Vasconsuelo et al., 2010). Furthermore, the ability 17β-estradiol treatment to block hydrogen peroxide induced C2Cl2 cell apoptosis was inhibited when HSP25 expression was blocked with siRNA (Vasconsuelo et al., 2010). However, the complete loss of HSP25 *in vivo* had no effect on the substantial increase in muscle tissue caspase-3 activity in old compared to young mice, indicating that while caspase-3 is an important factor in muscle mass decline, HSP25 likely plays no role in regulating this pathway.

In conclusion, this is the first *in vivo* report associating the specific absence of HSP25 with functional decrements in muscle contractile function and running wheel activity. Further, we also demonstrate that the absence of HSP25 has age-dependent effects in particular with running wheel activity. The dramatic difference in running between genotypes in young groups with no differences in the old groups suggests that the loss of HSP25 with aging likely has functional consequences. The greater difference between genotypes in running wheel activity may be due in part to the greater demands of this activity on multiple physiological systems as compared to measurements of isolated skeletal muscle fatigability. The increased fatigability and rate of force loss in young *H*sp25^−/−^ mouse muscle compared to *H*sp25^+/+^ suggests, however, that at least one mechanism for the reduced running wheel activity is greater skeletal muscle fatigability. We also report that activation of caspase-3 likely contributes to age-associated reductions in myofibrillar proteins and muscle mass. However, in contrast to *in vitro* findings, the absence of HSP25 had no significant effect on caspase-3 activity in skeletal muscle, *in vivo.*

### Conflict of interest statement

The authors declare that the research was conducted in the absence of any commercial or financial relationships that could be construed as a potential conflict of interest.

## References

[B1] Allen D. L. Harrison B. C. Maass A. Bell M. L. Byrnes W. C. Leinwand L. A. (2001). Cardiac and skeletal muscle adaptations to voluntary wheel running in the mouse. J. Appl. Physiol. 90, 1900–1908 10.1152/japplphysiol.00832.2001 11299284

[B2] Beere H. M. (2005). Death versus survival: functional interaction between the apoptotic and stress-inducible heat shock protein pathways. J. Clin. Invest. 115, 2633–2639 10.1172/JCI26471 16200196 PMC1236700

[B3] Concannon C. G. Gorman A. M. Samali A. (2003). On the role of Hsp27 in regulating apoptosis. Apoptosis 8, 61–70 10.1023/A:1021601103096 12510153

[B4] Du J. Wang X. Miereles C. Bailey J. L. Debigare R. Zheng B. (2004). Activation of caspase-3 is an initial step triggering accelerated muscle proteolysis in catabolic conditions. J. Clin. Invest. 113, 115–123 10.1172/JCI18330 14702115 PMC300763

[B5] Escobedo J. Pucci A. M. Koh T. J. (2004). HSP25 protects skeletal muscle cells against oxidative stress. Free Radic. Biol. Med. 37, 1455–1462 10.1016/j.freeradbiomed.2004.07.024 15454285

[B6] Feasson L. Stockholm D. Freyssenet D. Richard I. Duguez S. Beckmann J. S. (2002). Molecular adaptations of neuromuscular disease-associated proteins in response to eccentric exercise in human skeletal muscle. J. Physiol. 543, 297–306 10.1113/jphysiol.2002.018689 12181300 PMC2290467

[B7] Golenhofen N. Perng M. D. Quinlan R. A. Drenckhahn D. (2004). Comparison of the small heat shock proteins alphaB-crystallin, MKBP, HSP25, HSP20, and cvHSP in heart and skeletal muscle. Histochem. Cell Biol. 122, 415–425 10.1007/s00418-004-0711-z 15480735

[B8] Huey K. A. (2006). Regulation of Hsp25 expression and phosphorylation in functionally overloaded rat plantaris and soleus muscles. J. Appl. Physiol. 100, 451–456 10.1152/japplphysiol.01022.2005 16223977

[B9] Huey K. A. Burdette S. Zhong H. Roy R. R. (2010). Early response of heat shock proteins to functional overload of the soleus and plantaris in rats and mice. Exp. Physiol. 95, 1145–1155 10.1113/expphysiol.2010.054692 20851858

[B10] Huey K. A. McCall G. E. Zhong H. Roy R. R. (2007). Modulation of HSP25 and TNF-α during the early stages of functional overload of a rat slow and fast muscle. J. Appl. Physiol. 102, 2307–2314 10.1152/japplphysiol.00021.2007 17379754

[B11] Huey K. A. Meador B. M. (2008). Contribution of IL-6 to the Hsp72, Hsp25, and alphaB-crystallin [corrected] responses to inflammation and exercise training in mouse skeletal and cardiac muscle. J. Appl. Physiol. 105, 1830–1836 10.1152/japplphysiol.90955.2008 18927263 PMC2612468

[B12] Huey K. A. Roy R. R. Zhong H. Lullo C. (2008). Time-dependent changes in caspase-3 activity and heat shock protein 25 after spinal cord transection in adult rats. Exp. Physiol. 93, 415–425 10.1113/expphysiol.2007.041228 18156166

[B13] Huey K. A. Thresher J. S. Brophy C. M. Roy R. R. (2004). Inactivity-induced modulation of Hsp20 and Hsp25 content in rat hindlimb muscles. Muscle Nerve 30, 95–101 10.1002/mus.20063 15221884

[B14] Inaguma Y. Goto S. Shinohara H. Hasegawa K. Ohshima K. Kato K. (1993). Physiological and pathological changes in levels of the two small stress proteins, HSP27 and alpha B crystallin, in rat hindlimb muscles. J. Biochem. 114, 378–384 8282729 10.1093/oxfordjournals.jbchem.a124184

[B15] Jiang B. Xiao W. Shi Y. Liu M. Xiao X. (2005). Heat shock pretreatment inhibited the release of Smac/DIABLO from mitochondria and apoptosis induced by hydrogen peroxide in cardiomyocytes and C2C12 myogenic cells. Cell Stress Chaperones 10, 252–262 10.1379/CSC-124R.1 16184770 PMC1226023

[B16] Karakelides H. Sreekumaran Nair K. (2005). Sarcopenia of aging and its metabolic impact. Curr. Top. Dev. Biol. 68, 123–148 10.1016/S0070-2153(05)68005-2 16124998

[B17] Koh T. J. (2002). Do small heat shock proteins protect skeletal muscle from injury? Exerc. Sport Sci. Rev. 30, 117–121 10.1097/00003677-200207000-00005 12150570

[B18] Koh T. J. Escobedo J. (2004). Cytoskeletal disruption and small heat shock protein translocation immediately after lengthening contractions. Am. J. Physiol. Cell Physiol. 286, C713–C722 10.1152/ajpcell.00341.2003 14627610

[B19] Larkins N. T. Murphy R. M. Lamb G. D. (2012). Absolute amounts and diffusibility of HSP72, HSP25, and alphaB-crystallin in fast- and slow-twitch skeletal muscle fibers of rat. Am. J. Physiol. Cell Physiol. 302, C228–C239 10.1152/ajpcell.00266.2011 21975426

[B20] Marzetti E. Leeuwenburgh C. (2006). Skeletal muscle apoptosis, sarcopenia and frailty at old age. Exp. Gerontol. 41, 1234–1238 10.1016/j.exger.2006.08.011 17052879

[B21] McArdle A. Dillmann W. H. Mestril R. Faulkner J. A. Jackson M. J. (2004). Overexpression of HSP70 in mouse skeletal muscle protects against muscle damage and age-related muscle dysfunction. FASEB J. 18, 355–357 10.1096/fj.03-0395fje 14688209

[B22] McClung J. M. Kavazis A. N. Deruisseau K. C. Falk D. J. Deering M. A. Lee Y. (2007). Caspase-3 regulation of diaphragm myonuclear domain during mechanical ventilation-induced atrophy. Am. J. Respir. Crit. Care Med. 175, 150–159 10.1164/rccm.200601-142OC 17082496 PMC1899279

[B23] Meador B. M. Huey K. A. (2009). Glutamine preserves skeletal muscle force during an inflammatory insult. Muscle Nerve 40, 1000–1007 10.1002/mus.21430 19705479

[B24] Meador B. M. Huey K. A. (2011). Statin-associated changes in skeletal muscle function and stress response after novel or accustomed exercise. Muscle Nerve 44, 882–889 10.1002/mus.22236 22102458

[B25] Morton J. P. Holloway K. Woods P. Cable N. T. Burniston J. Evans L. (2009). Exercise training-induced gender-specific heat shock protein adaptations in human skeletal muscle. Muscle Nerve 39, 230–233 10.1002/mus.21182 19058194

[B26] Morton J. P. MacLaren D. P. Cable N. T. Bongers T. Griffiths R. D. Campbell I. T. (2006). Time course and differential responses of the major heat shock protein families in human skeletal muscle following acute nondamaging treadmill exercise. J. Appl. Physiol. 101, 176–182 10.1152/japplphysiol.00046.2006 16565353

[B27] Murlasits Z. Cutlip R. G. Geronilla K. B. Rao K. M. Wonderlin W. F. Alway S. E. (2006). Resistance training increases heat shock protein levels in skeletal muscle of young and old rats. Exp. Gerontol. 41, 398–406 10.1016/j.exger.2006.01.005 16524679

[B28] Nishimura R. N. Sharp F. R. (2005). Heat shock proteins and neuromuscular disease. Muscle Nerve 32, 693–709 10.1002/mus.20373 15962334

[B29] Paulsen G. Lauritzen F. Bayer M. L. Kalhovde J. M. Ugelstad I. Owe S. G. (2009). Subcellular movement and expression of HSP27, alphaB-crystallin, and HSP70 after two bouts of eccentric exercise in humans. J. Appl. Physiol. 107, 570–582 10.1152/japplphysiol.00209.2009 19498098

[B30] Paulsen G. Vissing K. Kalhovde J. M. Ugelstad I. Bayer M. L. Kadi F. (2007). Maximal eccentric exercise induces a rapid accumulation of small heat shock proteins on myofibrils and a delayed HSP70 response in humans. Am. J. Physiol. Regul. Integr. Comp. Physiol. 293, R844–R853 10.1152/ajpregu.00677.2006 17522120

[B31] Siu P. M. Alway S. E. (2005). Age-related apoptotic responses to stretch-induced hypertrophy in quail slow-tonic skeletal muscle. Am. J. Physiol. Cell Physiol. 289, C1105–C1113 10.1152/ajpcell.00154.2005 15972839

[B32] Solaro R. J. Pang D. C. Briggs F. N. (1971). The purification of cardiac myofibrils with Triton X-100. Biochim. Biophys. Acta 245, 259–262 10.1016/0005-2728(71)90033-8 4332100

[B33] Thomason D. B. Herrick R. E. Surdyka D. Baldwin K. M. (1987). Time course of soleus muscle myosin expression during hindlimb suspension and recovery. J. Appl. Physiol. 63, 130–137 2957349 10.1152/jappl.1987.63.1.130

[B34] Vasconsuelo A. Milanesi L. Boland R. (2010). Participation of HSP27 in the antiapoptotic action of 17beta-estradiol in skeletal muscle cells. Cell Stress Chaperones 15, 183–192 10.1007/s12192-009-0132-y 19621276 PMC2866980

[B35] Vasilaki A. Jackson M. J. McArdle A. (2002). Attenuated HSP70 response in skeletal muscle of aged rats following contractile activity. Muscle Nerve 25, 902–905 10.1002/mus.10094 12115981

[B36] Vasilaki A. McArdle F. Iwanejko L. M. McArdle A. (2006). Adaptive responses of mouse skeletal muscle to contractile activity: the effect of age. Mech. Ageing Dev. 127, 830–839 10.1016/j.mad.2006.08.004 16996110

[B37] Welsh M. J. Gaestel M. (1998). Small heat-shock protein family: function in health and disease. Ann. N.Y. Acad. Sci. 851, 28–35 10.1111/j.1749-6632.1998.tb08973.x 9668602

